# Management of unusual genital lymphedema complication after Fournier’s gangrene: a case report

**DOI:** 10.1186/1471-2482-12-26

**Published:** 2012-12-23

**Authors:** Oanna Meyer Ganz, Raphaël Gumener, Pascal Gervaz, Julien Schwartz, Brigitte Pittet-Cuénod

**Affiliations:** 1 Division of Plastic, Reconstructive and Aesthetic Surgery, Geneva University Hospitals and Medical School, Rue Gabrielle-Perret-Gentil 4, 1211 Geneva 14, Geneva, Switzerland; 2 Department of Surgery, Geneva University Hospitals and Medical School, Rue Gabrielle-Perret-Gentil 4, 1211 Geneva 14, Geneva, Switzerland; 3 Division of Urology, Geneva University Hospitals and Medical School, Rue Gabrielle-Perret-Gentil 4, 1211 Geneva 14, Geneva, Switzerland

**Keywords:** Fournier’s gangrene, Penile lymphedema, Negative pressure wound therapy, Perineal reconstruction

## Abstract

**Background:**

Fournier’s gangrene is a bacterial infection characterized by necrotizing fasciitis, skin and soft tissue involvement, and eventually myositis of the perineal region. Aggressive debridement of devitalized tissue and overlying skin is of paramount importance, but often leaves large defects to be reconstructed. The present case reports successful extensive perineal defects coverage following Fournier’s gangrene and management of subsequent penile lymphoedema impairing sexual function in a young patient.

**Case presentation:**

Following perianal abscess drainage, a healthy young man presented with scrotal pain. Fournier’s gangrene was diagnosed and treated with multiple surgical debridements. Tissue excision extended through the entire perineal area, base of the penile shaft, lower abdominal region, the inner thighs, and gluteal region, corresponding to 12% of the total body surface area. After serial debridements and negative pressure dressings, the defect was covered by two stages of skin grafting. Graft take was 90%. Healing was achieved without hypertrophic or retractile scar. However, chronic penile lymphedema remained and was first treated with compressive garments for 2 years. Upon failure of this conservative approach, we performed a circumcision, but only a “penile lift” allowed a satisfactory esthetical and functional result.

**Conclusion:**

Fournier’s gangrene can be complicated by a chronic lymphedema of the penis. Conservative treatment is likely to fail in severe cases and can be treated surgically by “penile lift”.

## Background

Fournier’s gangrene is characterized by necrotizing bacterial fasciitis and infection of soft tissue and skin of the perineal region [1]. Patients with Fournier’s gangrene may reveal pre-existing immune suppression of various conditions, but the disease also affects healthy individuals. Clinical onset is often insidious with minimal cutaneous lesions but typically progresses along deep fascial planes into a rapidly spreading sepsis with a potential fatal outcome in 3% to 45% of cases [2-4].

In contrast to necrotizing fasciitis of the extremities, many organisms can be involved; the combination of anaerobes and aerobes are the rule rather than the exception. However, as for all sorts of necrotizing fasciitis, beta-hemolytic *streptococci* of group A (*Streptococcus pyogenes*) are the most common causative pathogens [1].

Infection is rapidly invasive within hours despite antibiotic coverage, partly because antibiotic agents have difficulties to penetrate into destructed tissue with breakdown of blood supply due to formation of microthrombi, the histological hallmark of necrotizing fasciitis.

Hemodynamic support and parenteral broad-spectrum antibiotics are required to control severe sepsis, but prompt surgical debridement of all devitalized tissue is the mainstay of treatment. Tissues that can easily be divided from the fascial planes by digital dissection must be completely removed. Therefore, wide debridement is required, which leaves large defects to be covered [4-6]. Reconstruction is challenging due to humidity, contamination and irregularity of the perineal area.

## Case presentation

A healthy 33-year-old man arrived at the emergency department complaining of fever and scrotal pain, 3 days after para-anal abscess drainage. Physical findings included scrotal and perianal swelling, ischemic skin and crepitus over distal scrotum. Rapid progression to septic shock required hemodynamic support and ventilation and allowed diagnosis of Fournier’s gangrene.

**Figure 1:**
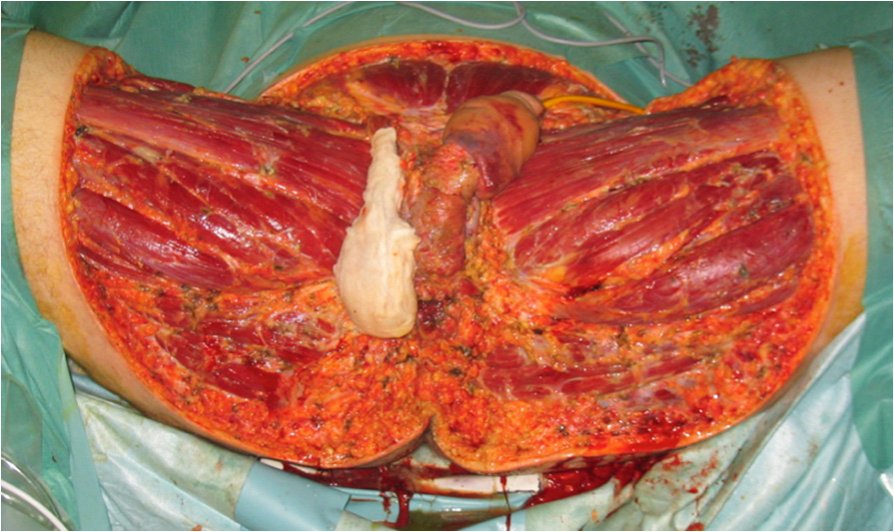
**Debridement on day 6.** Soft tissue defect corresponding to 12% of total body surface area. Right scrotum wrapped in gauze.

Urgent aggressive debridement on day 0 had to be repeated several times due to shock persistence and multiple organ failure. The consequent defect corresponded to 12% of the body surface and involved most of the perineal area including the base of the penis (Figure 1). Orchiectomy of a necrotic testis was performed and a diverting colostomy prevented wound contamination.

*Streptococcus pyogenes* was the main bacteria isolated from the wound. The histologic examination diagnosed necrotizing dermohypodermitis and vascular thrombi characterizing necrotizing fasciitis.

**Figure 2:**
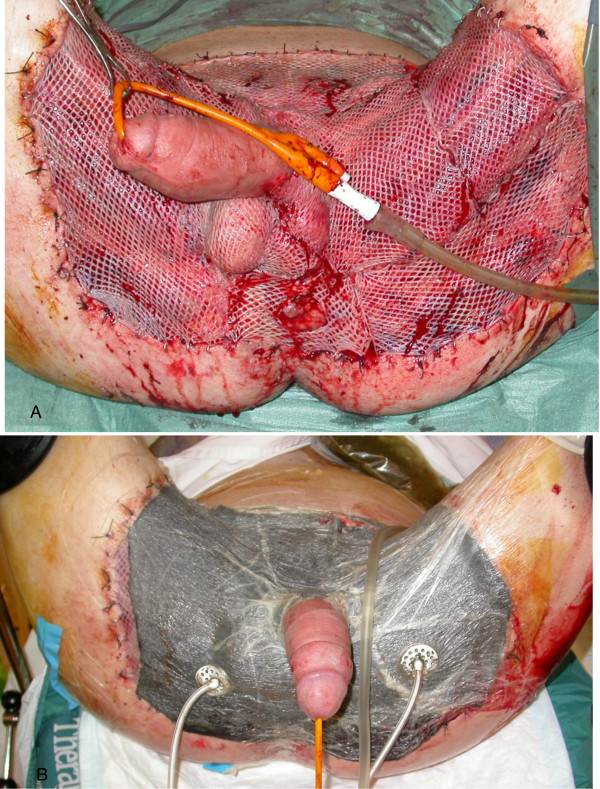
Skin grafting (A) and skin grafting covered by vacuum-assisted closure dressing (B).

Negative pressure dressings were applied on day 6 and changed regularly to prepare the wound bed for grafting. The abdominal and perineal defects were then covered by meshed split skin grafts in 2 steps and vacuum-assisted closure (VAC®) was used for dressing (Figure 2).

**Figure 3:**
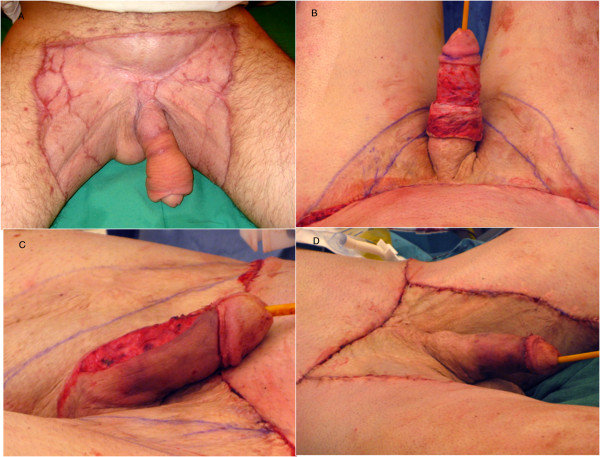
**Six months after skin grafts.** No scar retraction but penile lymphedema was persistent **(A)**. The penile lift **(B,C,D)**.

Overall graft take was 90%. Neither hypertrophic nor retractile scar was detected during follow-up (Figure 3A).

### Long-term follow-up

Complete anodermal destruction resulted in anal stenosis. Anoplasty by cutaneous advancement flap in the grafted area was successfully carried out 18 months later.

The patient suffered from testicular pain when sitting because the remaining grafted testis was fixed over the perineum. Neoscrotal plasty was therefore performed 2 years later with local skin flaps in the grafted skin region.

One of the main concerns of this young man was persistent penile lymphedema, skin excess and altered sensitivity over the distal penile shaft, which affected his sex life (Figure 3A). After unsuccessful conservative treatment for 2 years we performed a circumcision with a large excision of skin excess along with resection of the underlying subcutaneous edematous tissue. However, lymphedema persisted in the remaining distal penile shaft and the patient was very demanding for additional correction despite the risks of worsening the hypoesthesia. Therefore, in a second step, we undertook a “penile lift,” consisting of a circumferential incision around the coronal sulcus and a ventral incision through the median raphe. The skin was lifted, degloving the penile shaft to 3 centimeters from the skin graft located at the base. This proximal bridge of penile skin adjacent to the skin graft was not edematous and did not need subdermal excision; it therefore provided the vascular supply for the cutaneous flap. Radical subcutaneous excision removed all fibrotic and edematous tissue lying above Buck’s fascia. The dermocutaneous flap was pulled down over the penile body, and the distal skin excess was excised (Figure 3B-D). The flap edges looked well vascularized. At the same time, we performed a partial scar removal by abdominal and crural lift to reduce the grafted surface.

**Figure 4:**
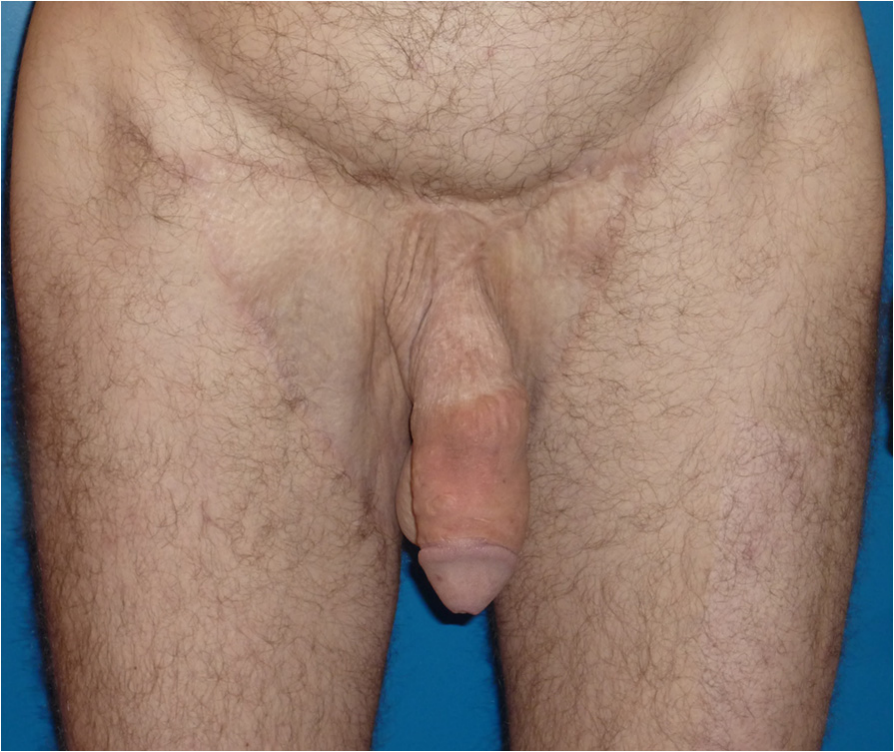
**Twenty months following the last operation.** No hypertrophic scar and no lymphoedema reccurence.

Sensitivity of the penile shaft remained diminished but glans sensitivity was normal and no recurrence of lymphedema was observed. The patient now has normal mobility. He is very satisfied with the outcome and has returned to a normal professional, social, and sexual life (Figure 4).

## Conclusions

This case of severe Fournier’s gangrene challenged us with two main problems: a) the existence of a particularly large perineal soft tissue defect and b) a voluminous chronic penile shaft lymphedema. Options for repairing perineal defects include fasciocutaneous flaps for small surface defects and skin grafts for large defects [5-7]. Flaps are preferred because their use prevents graft maceration and scar retraction. Skin grafting is a simple and well-adapted technique for extended skin defect [5,8]; however, in perineal area partial graft take is the rule, and hypertrophic or retractile scar can occur by secondary healing, leading to functional limitations.

Using vacuum-assisted therapy in a humid and irregular area effectively cleans and prepares the wound [9,10] for skin grafting. The negative pressure aspirates liquids from the wound, thereby reducing humidity and bacterial load. The mechanical suction also promotes granulation tissue formation by stimulating angiogenesis and myofibroblast proliferation [9]. Moreover, by increasing graft survival rate and decreasing the time necessary to achieve wound healing, it helps in reducing hypertrophic scarring and secondary retraction as demonstrated in our patient.

The second complication of our Fournier’s gangrene case was penile lymphedema with functional impairment during intercourse and cosmetic embarrassment.

In 1820, Delpech described a case of scrotal edema successfully treated by excising all the lymphedematous tissue [11]. Variations of Delpech’s original technique involve excision of the penile skin and subcutaneous tissue containing the superficial lymphatics at the level of Buck’s fascia, followed by coverage with skin graft or local flaps. This treatment is widely used for penoscrotal elephantiasis and provides satisfying long-term results. In our case, tissue over the distal penile shaft was not affected by the initial infection. Circumferential debridement of the penile skin and subcutaneous tissue was therefore limited to the penile base, impairing superficial lymphatic flow of the distal penis. In contrast to penoscrotal elephantiasis, the skin overlying the lymphedematous tissue in the present case exhibited no changes. Therefore, instead of resecting the skin with the affected subcutaneous tissue, we decided to preserve the residual healthy penile skin and to use it as a flap for coverage despite the risk of skin devascularization: the blood supply came from the bridge of undegloved penile skin adjacent to the skin graft at the penile base. This approach avoided a skin graft over the penile shaft, which would lose the necessary characteristics of penile skin as softness, elasticity and mobility. In conclusion, Fournier's gangrene spread to male genital organs presents therapeutic challenges; a multidisciplinary collaboration involving plastic surgeons at the time of initial surgical debridement is required for optimal wound management and to lower risks of retractile scarring.

### Consent

Written informed consent was obtained from the patient for publication of this Case report and any accompanying images. A copy of the written consent is available for review by the Editor of this journal.

## Competing interests

The authors declare that they have no competing interests.

## Authors’ contributions

OMG, RG and BPC acquired the data and wrote/revised the manuscript. OMG and BPC performed the clinical follow-up of the patient. OMG, BPC, PG and JS performed the surgery. Furthermore, all authors have been involved in revising the manuscript critically for important intellectual content read and approved the final manuscript.

## Pre-publication history

The pre-publication history for this paper can be accessed here:

http://www.biomedcentral.com/1471-2482/12/26/prepub
